# Leisure Engagement during COVID-19 and Its Association with Mental Health and Wellbeing in U.S. Adults

**DOI:** 10.3390/ijerph19031081

**Published:** 2022-01-19

**Authors:** Xiangyou Shen, Megan MacDonald, Samuel W. Logan, Colby Parkinson, Lydia Gorrell, Bridget E. Hatfield

**Affiliations:** 1 Department of Forest Ecosystems and Society, College of Forestry, Oregon State University, Corvallis, OR 97331, USA; colby.parkinson@oregonstate.edu (C.P.); lydia.gorrell@oregonstate.edu (L.G.); 2 College of Public Health and Human Sciences, Oregon State University, Corvallis, OR 97331, USA; Megan.MacDonald@oregonstate.edu (M.M.); Sam.Logan@oregonstate.edu (S.W.L.); Bridget.Hatfield@oregonstate.edu (B.E.H.)

**Keywords:** COVID-19, wellbeing, pandemic, sustained stress, stress alleviation, physical activity, nature-based activity, digital leisure, leisure engagement, subjective wellbeing

## Abstract

Leisure engagement has risen as a salient societal issue during the COVID-19 pandemic, not only because it provides a pathway for people to continue meeting their physical, cognitive, and social-emotional needs, but also due to the phenomenal juxtaposition of general increases in leisure time and unparalleled constraints. This study reports the results of the first investigation of U.S. adults’ overall leisure engagement and its association with mental health amidst the major disruptions and sustained stress of the COVID-19 pandemic. Qualitative and quantitative data were collected through an online survey in February 2021 through Prolific from a sample representative of the U.S. adult population in age, gender, and race (*n* = 503) and analyzed using a mixed-method approach. A total of 104 unique leisure activities in 19 categories and 3 domains were identified through iterative thematic coding. Participants reported general increases in home-based traditional leisure and digital/online activities and decreases in physical and nature-based activities. Multiple regression analyses controlling for socio-demographic and context-specific covariates revealed distinct associations between changes in leisure engagement and different aspects of mental health (perceived stress, depressive symptoms, and mental wellbeing), supporting leisure’s dual role in facilitating stress alleviation and wellbeing enhancement during taxing events, such as COVID-19.

## 1. Introduction

The COVID-19 pandemic has had an enormous impact on societies worldwide, causing major disruptions to people’s everyday life. Restrictive public health measures to curb the spread of COVID-19, such as social distancing and stay-at-home orders, not only increased the risk for poor mental health [1,2,3], but also placed unprecedented constraints on what people could do to maintain a good quality of life. While the majority of COVID-19 studies on mental health emphasized risk factors, vulnerabilities, and adverse psychiatric outcomes [4], a growing number of studies have investigated mechanisms that may assist with coping and enhance resilience [5,6,7,8]. Leisure is one such pathway through which people may continue to fulfill their physical, cognitive, and social-emotional needs while dealing with the challenges of COVID-19 [9]. 

Longstanding research has highlighted leisure’s positive contribution to wellbeing [10] through multiple pathways, from providing time structure [11] to fulfilling fundamental psychological needs, such as autonomy [12], mastery [13], affiliation [14], restoration [15], and meaning [16,17] (see [18,19] for reviews). In particular, leisure’s therapeutic effect in buffering the negative impact of stressful situations has been well documented [20]. Empirical studies have examined leisure’s utility in coping with daily hassles and normative life stressors [21,22], chronic stress and work stress [23], life-altering events [24], and among people regularly under high stress [25]. Evidence suggests that leisure can be an important means through which people cope with stress and maintain good physical and mental health [23] and that leisure contributes to adaptive outcomes above and beyond the effects of general coping (e.g., problem-focused coping unrelated to leisure) [25]. 

In the context of the COVID-19 pandemic, maintaining engagement in valued leisure activities is believed to provide a protective benefit for mental health and help mitigate the adverse effect of sustained stress [9]. Leading health organizations such as the World Health Organization [26] and the Centers for Disease Control and Prevention [27] have advised ensuring time for leisure pursuits as a way of self-care during the pandemic. A number of commentary pieces have also emphasized the importance of examining leisure engagement during COVID-19 and offered predictions of possible changes or anecdotal observations [9,28,29,30]. However, limited empirical studies have examined how leisure engagement may have changed during the pandemic and whether its maintenance, or a lack thereof, affects mental health and psychological wellbeing. Despite the generally positive relation between leisure engagement and long-term mental health observed in past studies [10], it is unclear that in times of major disruptions whether higher levels of stress would trigger greater participation in leisure (i.e., a positive relationship) or if stress would reduce as a result of adaptive increases in leisure engagement (i.e., a negative relationship). The present study addresses these questions by characterizing the broad trends in people’s leisure engagement during the height of COVID-19 and its relationship with mental health and wellbeing. This study was conducted in the United States (U.S.), one of the most affected countries that currently leads the world in the cumulative numbers of confirmed cases (59.4 million as of 8 January 2022) and ranked 5th in the number of deaths (254.9 per 100,000 people, surpassed only by Bulgaria, Hungary, Czechia, and Poland) among the twenty most affected countries by COVID-19 worldwide [31]. A brief review of the context and existing research on the topic follows below.

Since the CDC confirmed the first COVID-19 case in the U.S. on 21 January 2020 and the World Health Organization officially declared the COVID-19 outbreak a pandemic on 11 March 2020 [26], the U.S. pandemic journey has evolved through many phases as confirmed COVID cases and deaths fluctuated, new variants arrived, pandemic fatigue settled in, and vaccination rates and recommendations on social distancing, masking, and quarantine continue to evolve [32]. As in many other parts of the world, the U.S. registered heightened mental health strain, with the percentages of adults reporting anxiety or depressive symptoms nearly quadrupling (from 1 in 10 to 4 in 10) over the course of 2020 and staying high throughout 2021 [33]. 

Associated with the increased risk for poor mental health were lifestyle changes as a result of social distancing and stay-at-home orders, which have seen, among other trends, an increasing number of people working from home and more leisure time for many. The American Time Use Survey (ATUS) by the U.S. Bureau of Labor Statistics (BLS) reported that the percent of people working at home doubled during the pandemic in 2020, rising to 42%. Meanwhile, the average time spent in leisure and sports activities increased by 32 min per day, partly due to the widespread decline in time spent traveling, and the reduction in average work time as a result of the decreased employment rate during the pandemic [34]. 

The additional leisure time coincided with unprecedented constraints in the form of movement restrictions, the complete elimination or drastic reduction of large social gatherings, and the closure or reduced operation of public and private leisure facilities (e.g., gyms, swimming pools, museums, bars, restaurants, and parks at some point in many places) [35,36]. Researchers have noted a revaluing of “traditional” forms of at-home leisure, such as reading, board games, gardening, and house chores [29], and a spike in the popularity of gaming, streaming, and social-media-based activities [37,38]. Meanwhile, empirical studies and systematic reviews reported uneven decreases in physical activity [39,40,41] and mixed findings on time spent outdoors [35]. However, the majority of existing empirical studies (a) reported changes in early stages of the pandemic (shortly following or within 6–9 months of the outbreak), (b) were conducted outside of the U.S. [37,42], or included U.S. data but did not provide a country-specific analysis [43], (c) focused on specific types of leisure behavior, predominantly leisure-time physical activities [44,45,46,47,48] and outdoor recreation [49,50,51,52], and/or (d) examined leisure independent of risk factors and did not account for confounding health variables, such as general health and enduring psychological wellbeing [43]. Thus, while many valuable insights were gained from recent empirical work, much remains unknown about the broad pattern of leisure engagement in the U.S. and its role in shaping wellbeing relative to context-specific risk factors during COVID-19.

The present study fills the above knowledge gaps by examining (a) people’s engagement in leisure under sustained stress beyond the initial stages of COVID-19, and (b) the relations between leisure engagement and mental health among U.S. adults through a national survey conducted at the height of the pandemic. We seek to answer the following two questions:

1. What is the general pattern of leisure engagement during COVID-19 among U.S. adults? Specifically, what are the types of leisure activities, frequency of participation, changes in engagement level, and people’s subjective evaluations of their engagement?
2. How is leisure engagement associated with mental health and wellbeing, above and beyond the effect of potential confounding variables, such as COVID-specific risk factors, social-demographic backgrounds, general physical health, and global psychological wellbeing?

## 2. Materials and Methods

### 2.1. Study Design and Procedures

Cross-sectional data were collected through an online survey set up on Qualtrics [53] and distributed through Prolific [54], an online crowdsourcing research platform. Past studies suggested that Prolific compared favorably to alternative crowdsourcing platforms (e.g., Amazon Mechanical Turk and CrowdFlower) with respect to data quality and participant diversity [55,56]. The present study utilized the platform’s built-in pre-screening technique to ensure that participants were (1) at least 18 years old, (2) residing in the U.S. at the time of survey, and (3) representative of the U.S. adult population on age, gender, and race. 

A total of 505 participants responded to the survey between 3 and 15 February 2021. Two cases were removed due to large numbers of missing values as a result of drop-out attrition, yielding a sample size of 503. In the same survey, other aspects were investigated as part of a larger project on leisure and coping. The study was approved by the Institution Review Board of the Oregon State University (IRB-2020-0927). All participants provided consent before completing the survey. The survey took 15–20 min to fill out and each participant received monetary compensation at a “fair pay” rate as recommended by the platform to ensure data quality [57].

### 2.2. Materials and Measures

#### 2.2.1. Mental Health Outcomes

##### Perceived Stress 

Perceived stress was measured using the 7-item stress sub-scale from the Depression, Anxiety, and Stress Scale (DASS-21) [58]. DASS-21 has been validated in both clinical [59] and nonclinical samples [60] and used in several COVID-19 studies [61,62]. The stress sub-scale assesses difficulty relaxing (e.g., “I found it hard to wind down”), nervous arousal and being easily agitated, upset, irritable or over-reactive (e.g., “I found myself getting agitated”), and impatient (e.g., “I was intolerant of anything that kept me from getting on with what I was doing”). Items are rated on a 4-point scale ranging from 0 (“did not apply to me at all”) to 3 (“applied to me most of the time or always”). The internal consistency of this subscale was excellent in the current study (Cronbach’s α = 0.92). A total stress score ranging from 0 to 21 was calculated by summing the seven item scores.

##### Depressive Symptoms

Depressive symptoms were measured using the brief Patient Health Questionnaire (PHQ-2), a depressive symptoms screening tool with established reliability and validity [63]. The scale consists of two items designed to assess the past-two-week frequency of experiencing two cardinal symptoms of depression: depressed mood (“little interest or pleasure in doing things”) and anhedonia (“feeling down, depressed, or hopeless”). Responses were recorded using a four-point scale: 0 = “not at all”, 1 = “several days”, 2 = “more than half the day”, and 3 = “nearly every day”. The scale showed good internal consistency in the current sample (Cronbach’s α = 0.85). Responses were summed to yield a total score ranging from 0 to 6. 

##### Mental Wellbeing

Mental wellbeing was measured with the 5-item World Health Organization Well-being Index [64], a short, generic global rating scale designed to tap a respondent’s recent experience in positive mood (e.g., “I have felt cheerful in good spirits”), vitality (e.g., “I have felt active and vigorous”), and general interests (e.g., “my daily life has been filled with things that interest me”) over the previous two weeks. Items were measured with a six-point scale ranging from 0 (“at no time”) to 5 (“all of the time”). The scale has been widely used and demonstrated adequate psychometric properties [65]. It showed high internal consistency (Cronbach’s α = 0.92) in the current study. A mental wellbeing index ranging from 0 to 25 was created by summing the responses to the five items. 

#### 2.2.2. Leisure Engagement

Past systematic review suggested that frequency and diversity measures of leisure engagement are more strongly associated with psychological wellbeing than measures of time spent on leisure [10]. We measured leisure frequency, variety, and engagement relative to the pre-COVID period and participants’ desired level. Self-identified *valued leisure activities* were emphasized for two reasons: (a) people were most motivated to pursue valued leisure activities in the face of constraints, and (b) engagement in these activities, or a lack thereof, was most likely to have an impact on people’s mental health and psychological wellbeing. 

Specifically, participants were asked to identify their “favorite leisure activity” by naming the one activity that they enjoyed the most during the pandemic, followed by questions on (1) the frequency of engaging in the identified activity during the pandemic (5-point scale: 1 = “less than once a week” to 5 = “Almost every day”), (2) perceived change in engagement relative to the pre-COVID level (5-point scale: 1 = “much less…” to 5 = “much more …”; an additional option of “cannot compare—this is a new activity I started during the pandemic” was included to gauge the prevalence of starting a new valued activity during COVID-19), and (3) evaluation of the current engagement level relative to one’s “ideal” level (3-point scale: 1 = “much less than I would like”, 2 = “a little less than I would like”, and 3 = “about right”). 

#### 2.2.3. Contextual and Socio-Demographic Covariates

Three types of covariates were included in the survey to account for the potential confounding effects of (1) COVID-specific risk and protective factors, (2) socio-demographics and COVID-specific background variables, and (3) general health and psychological wellbeing. 

##### COVID-Specific Risk and Protective Factors 

Participants completed several questions regarding their perceptions, beliefs, and behaviors related to COVID-19 that might serve as risk or protective factors, each recorded on a 6-point Likert scale (1 = “Disagree strongly” and 6 = “Agree strongly”). 

Perceived risk of infection was assessed by asking the participant to rate “the likelihood of acquiring COVID-19 in general”. 

To address the potential confounding effect of optimism as a stress buffer to wellbeing [66], seven questions were developed for this study to assess (1) positive beliefs about public health preventative measures (two items: “…help lower the risk of infection”; “…help make our environment safer”), (2) negative beliefs about these preventative measures (three items: “…are constraining”, “…cause anxiety in me”, and “…make it hard to live a full life”), and (3) future outlook (two items: “Situations are improving with the development of COVID-19 vaccines”; “I am optimistic that life will return to normal soon”). The internal consistency of the above three sets of items were adequate (Cronbach’s α = 0.82, 0.73, and 0.61, respectively). Average scores were used for each scale. 

One global question assessing safe health behavior (“I take active precautionary measures to lower the risk of infection”) was included to control for its potential impact on leisure pursuit and countering effect on risk factors [5,67].

##### Socio-Demographics and COVID-Related Background 

We collected information on age (in years), gender (male, female, or trans/non-binary), race (five categories recoded into four: White, Asian, Black or African American, and other), ethnicity (Spanish, Hispanic, Latino or not), education attainment (high school or less, some college, college, and post-graduate), family income (<$29,999, $30,000 to $69,999, $70,000 to $99,999, or $100,000 or above), subjective financial situation (1 = “I’m behind on my bills”, 2 = “I’m barely making ends meet”, 3 = “I’m just getting by”, 4 = “I’m doing alright”, 5 = “I’m living comfortably”), marital status (married, never married, or divorced/separated/widowed), current work status (working from home, working outside home-high-risk position, working outside home-non-high-risk position, or unemployed/furloughed), and living area (rural to town [<5000 population], small city [5000–50,000 population], or medium or large city [>50,000 population]). Parenting status (yes [including being a legal guardian] or no) was also included to control for the potential effect of parenting on leisure engagement and perceived stress [68]. 

Three additional single-item questions were used to record COVID-related background: infection status (confirmed, suspected, or neither confirmed nor suspected), vaccination status (no, partially vaccinated, or fully vaccinated), and preexisting medical conditions (yes or no).

##### General Physical Health and Long-Term Subjective Wellbeing

General physical health and global subjective wellbeing (SWB) often remain relatively stable over a short to medium period of time and can be a source of resilience that buffers the negative impact of stressful life events [69,70]. Two measures were included to control for the effect of general physical health and SWB. 

General physical health was assessed with five general health perception questions in the RAND 36-Item Health Survey 1.0 [71]. Example items include “My health is excellent” and “I seem to get sick a little easier than other people” (reverse-worded). The scale showed adequate internal consistency (Cronbach’s α = 0.82) in the current study. Total general physical health scores (ranging from 0 to 100) were calculated following a two-step process per the RAND guideline. 

SWB was assessed using the Satisfaction with Life Scale (SWLS) [72], a 5-item global measure of subjective wellbeing. Example items include “In most ways my life is close to my ideal” and “so far I have gotten the important things I want in life” (7-point Likert scale: 1 = “strongly disagree”, 7 = “strongly agree”). Past studies reported good internal consistency and test-retest reliability [73]. High internal consistency (Cronbach’s α = 0.92) was observed in the current study. SWB scores ranging from 5 to 35 were calculated by summing the five item scores.

### 2.3. Data Analyses

#### 2.3.1. Data Processing and Inspection

The data were first inspected for missingness and assumptions. The diagnosis suggested a small proportion (0.46%, <2.5% per variable) of values were missing completely at random (Little’s MCAR test: χ^2^ = 56.51, df = 64, *p* = 0.736). Thus, complete cases were used in all subsequent analyses. We also verified that all model assumptions (e.g., normality, outliers, multicollinearity, and linearity) were met. One variable, change in engagement level, was treated as categorical in the regression analysis due to nonlinearity, as indicated by scatterplots.

#### 2.3.2. Sample Descriptive Analysis 

Participants’ characteristics in socio-demographics and COVID-related backgrounds were analyzed using descriptive analysis, including counts, proportions, means, and standard deviations, for each variable. A snapshot of mental health and wellbeing was also described by interpreting the summary scores in perceived stress, depressive symptoms, and mental wellbeing per the DASS guideline [74], the PHQ-2 guideline [75], and the WHO guideline [64], respectively.

#### 2.3.3. Leisure Activity Coding and Descriptive Analysis

To describe pandemic leisure engagement, we first analyzed the text responses to the open-ended question about “favorite leisure activity” and, through iterative coding and aggregation, identified the main categories of valued leisure activities based on shared features in forms, content, or resources involved (e.g., birdwatching, boating, fishing, and hiking were categorized as nature-based activities). The activity categories were then further grouped into one of three broad domains based on the main venue—home offline, online, or outdoor—where the activity took place. Listening to music, audiobooks, and podcasts, while involving digital content, did not rely on extensive interactions with a screen, and, therefore, were grouped under home-based offline activities. All board games (e.g., playing chess), card games (e.g., playing poker), and other games (e.g., jigsaw puzzles and crossword puzzles) were categorized as “playing games offline” unless “online” or “computer” was mentioned. Physical activity/sports and outdoor activities were grouped together to be consistent with common categorizations in previous research [43]. All activities were coded by two independent raters. Interrater agreement for the lower-level categorization was high (Cohen’s kappa = 0.95, *p* < 0.001). 

After activity categorization was finalized, we described engagement levels by different activity categories and domains and the type and frequency of valued leisure activities by socio-demographic characteristics. Descriptive analysis (e.g., counts, proportions, means, and standard deviation) and bivariate analyses (e.g., nonparametric tests and correlation analysis) were performed as appropriate. 

#### 2.3.4. Regression Analysis

To examine how leisure engagement related to mental health and wellbeing, three series of multiple regression analyses were conducted to test whether engagement in valued leisure activities (activity type, frequency, changes relative to the pre-COVID time, and evaluation of engagement level) predicted perceived stress, depressive symptoms, and mental wellbeing, independent of potential confounders. Each series of linear regression analyses first tested the unadjusted relationships (Model 1), then hierarchically adjusted for COVID-specific risk and protective factors (Model 2), socio-demographics and COVID-related background (Model 3), and general physical health and SWB (Model 4), each later model adding additional covariates to the previous model. 

We selected covariates based on a theoretical understanding of how each variable affects leisure engagement and mental health during the pandemic, balanced with considerations of model parsimony. Specifically, existing studies suggested that gender, age, civil/marital status, education, employment, living area, and presence of chronic/psychiatric illness significantly associated (with varying effect sizes) with mental health [4,76,77]. Moreover, younger people were more likely to engage in leisure time physical activity than older people [78]. Inconsistent patterns were reported about leisure engagement by other socio-demographic variables, suggesting that the relation between leisure participation and specific sociodemographic factors may vary across leisure activities and change over time [79,80].

The full model included nine covariates: five socio-demographic variables (age, gender, subjective financial conditions, marital status, and parenting), two COVID background variables (infection status and pre-existing conditions), and two global health and wellbeing variables (general physical health and SWB). A correlation matrix between all the variables in the analyses (Supplementary Material Figure S1) was obtained to examine their bivariate interrelationships (Pearson’s r for continuous variables and a point biserial correlation coefficient for dichotomous variables, with all categorical variables dummy coded). The change in engagement level was dummy-coded to reflect two groups: much less than pre-COVID and much more than pre-COVID (“a little less/a little more than pre-COVID” and “about the same” were combined to form an “about same” reference group). Vaccination status was not included, given the small number of fully vaccinated people in the study sample.

Results were considered statistically significant at *p* < 0.05. Cramer’s V, Pearson’s correlation (r), or the eta coefficient (η) were used to indicate the effect size of measures of association based on the variable type and interpreted based on the criteria suggested by Cohen (1988) [81]: small (0.10), medium (0.30), and large (0.50) for correlation coefficient r and Cramer’s V, and small (0.10), medium (0.24), and large (0.37) for eta. All analyses were run in IBM SPSS 27 (IBM Corp. IBM SPSS Statistics for Windows, Version 27.0. Armonk, NY, USA 2020.)

## 3. Results

### 3.1. Participants’ Characteristics

#### 3.1.1. Socio-Demographic Characteristics

Table 1 summarizes the descriptive data on socio-demographics and COVID-related background. The sample was representative of the U.S. adult population with respect to gender (50.7% female), age (mean = 46.6 ± 16.1 years, range = 20–79), and race (73.7% White). The majority of respondents were not Spanish, Hispanic, or Latino (92.8%), held a bachelor’s or higher degree (57.0%), worked from home (42.8%), had a household income less than $70, 000 (63.3%), were married (44.6%), not parenting (61.6%), and lived in a medium or large city with a population greater than 50,000 (51.6%). While almost half (49.7%) of the respondents reported doing alright financially or living comfortably, nearly 20% indicated they were barely making ends meet or behind on bills. 

#### 3.1.2. COVID-Related Background and a Snapshot of Mental Health

The majority of the respondents indicated having not been confirmed or suspected of COVID infection (87.7%) or vaccinated (91.8%) at the time of the survey. The percentage of people who received partial or full vaccination in the sample is comparable to the national rate of the same period (8.6% as of 5 February 2021) [82]. Over one third (38%) of the respondents reported having a chronic disease or pre-existing medical/psychiatric illness. 

While the majority of respondents (64%) reported a normal level of stress, near 30% reported mild or moderate stress and another 13% reported experiencing severe or extremely severe stress. About one-quarter (24%) of respondents reported major depressive symptoms and nearly 7% reported poor mental wellbeing. Details on participant mental health by socio-demographics are provided in the Supplementary Material Table S1.

### 3.2. Leisure Engagement during COVID-19

#### 3.2.1. Descriptive Patterns of Valued Leisure Activities 

A total of 104 unique activities were identified. After combining similar activities based on shared features in forms, content, or resources involved, a total of 19 categories emerged (Table 2), each falling into 1 of 3 broad domains based on the main activity venue (in the order of popularity): home-based offline activities (43.4%), screen-based digital/online activities (32.1%), and physical/outdoor activities (24.5%). 

Specifically, reading and/or writing were identified as a valued leisure activity by the largest proportion of people (18.9%), followed by playing video/online games (16.1%) and watching TV/movies/videos (13.9%). Notably, the majority of people were already engaged in their valued leisure activity before the pandemic; only a small proportion of respondents (2.4%) reported picking up a new favorite leisure activity during COVID-19. Because we only asked if people started a new activity that they also identified as their favorite activity, this number is likely lower than the proportion of people who tried a new leisure activity during the pandemic.

#### 3.2.2. Level of Engagement by Leisure Activity Categories and Domains

Table 2 also presents the self-reported frequency of engaging in valued leisure activity and the engagement level relative to pre-COVID level and one’s desired level. On average, engagement in listening to music, watching TV/movies/videos, playing video/online games, and reading and writing were the highest (M = 4.1–4.4, or more than 4–5 times a week), followed by walking, gardening, and playing games offline (M = 3.8–3.9, or slightly less than 4–5 times a week). Notably, people in field/court sports, nature-based activities, and traveling/driving reported the lowest level of participation (M = 1.3–1.6, or less than once a week). When comparing the current engagement level to the pre-COVID level, the majority of people reported an increase (M > 3, a value of 3 indicating maintaining the same level), including across-the-board increases in screen-based digital/online activities. Notably, while engagement in walking and gardening were comparable to pre-COVID level (M = 3), most other physical/outdoor activities saw a decrease in engagement (M < 3). When comparing the current engagement with the desired level, people engaged in cooking and baking, listening to music, relaxing, and all types of screen-based digital/online activities reported participating in these activities at a level close to their ideal (M = 2.6–2.9). The largest gap was observed in field/court sports (M = 1.1), traveling/driving (M = 1.2), and nature-based activities (M = 1.5).

Given the uneven group sizes across different activity categories and small occurrences in some, we compared engagement at the activity domain level. The results suggested significant differences in all three measures of engagement (*p* < 0.001). On average, people in screen-based digital/online activities reported the highest frequency of participation (M = 4.3, or more than 4–5 times a week), the largest increase in engagement relative to the pre-COVID level (M = 3.73), and were participating at a level closest to their ideal (M = 2.6). By contrast, people in the physical/outdoor activity category reported the lowest frequency (M = 2.7, or less than 2–3 times a week), decreased engagement compared to the pre-COVID time (M = 2.6), and the largest gap between the current engagement level and the desired level (M = 1.9). People in home-based offline activities reported an in-between level in all three measures, including participating in these activities close to 4–5 times a week (M = 3.9), which slightly exceeded their pre-COVID level (M = 3.3) and came close to their ideal (M = 2.4).

#### 3.2.3. Leisure Domain and Engagement Level by Socio-Demographic Characteristics

Table 3 shows the socio-demographic characteristics associated with the leisure domains and engagement levels during the pandemic. Several patterns are worth noting: (a) females were more likely to report a home-based offline activity as their valued leisure activity; males were more likely to indicate a screen-based digital/online activity and, to a lesser degree, a physical or outdoor activity (*p* < 0.001, Cramer’s V = 0.19); (b) people with a college or higher education were less likely to identify a screen-based digital/online activity, and, like people with a higher household income (*p* = 0.029, Cramer’s V = 0.12), more likely to identify a physical/outdoor activity as their favorite leisure (*p* = 0.004, Cramer’s V = 0.14); (c) on average, people in the digital/online activity category were about six years younger than those in the physical/outdoor activity category (*p* = 0.009, η = 0.14); and (d) non-parenting respondents reported slightly more frequent engagement than people who were parenting. No significant differences in leisure engagement level were observed between the other socio-demographic groups.

### 3.3. Leisure Engagement and Mental Health Outcomes

Table 4 presents the results of the fully adjusted linear regression models for all three outcome measures. The details of the unadjusted and intermediately adjusted models are provided in the Supplementary Material Tables S2–S4.

#### 3.3.1. Leisure Engagement and Perceived Stress

The results of the unadjusted model (see Supplementary Material Table S2) suggested that activity type, change in engagement level (relative to pre-COVID level), and self-evaluation of engagement (relative to the ideal level) were all significantly associated with perceived stress, while frequency was not. However, only increased leisure engagement predicted higher perceived stress (0.21 points higher than the maintenance group, *p* = 0.027) after adjusting for COVID-related risk and protective factors, socio-demographic and COVID-related background variables, and general physical health and SWB. Collectively, all leisure variables predicted 4% of the variance in perceived stress, with the full model explaining 36% of the total variance.

The result also suggested that (in the order of contribution) younger age (β = −0.44, *p* < 0.001), negative beliefs about preventative measures (β = 0.25, *p* < 0.001), poorer general physical health (β = −0.15, *p* = 0.003), lower life satisfaction (β = −0.10, *p* = 0.025), poorer subjective financial conditions (β = −0.10, *p* = 0.016), and higher perceived risk of infection (β = 0.10, *p* = 0.015) were associated with higher levels of stress.

#### 3.3.2. Leisure Engagement and Depressive Symptoms

The unadjusted model suggested that activity type, change in engagement level, and evaluation of engagement, but not frequency, predicted depressive symptoms (see Supplementary Material Table S3). After controlling for all three sets of covariates, only reduced level of engagement remained a significant predictor of higher depressive symptoms (0.61 higher than the maintenance group, *p* = 0.015). The explanatory power of leisure variables and the full model were similar to that of the perceived stress model. 

The full model also suggested that older age (β = −0.33, *p* < 0.001), better physical health (β = −0.24, *p* < 0.001), higher life satisfaction (β = −0.23, *p* < 0.001), and never married (relative to married, β = −0.13, *p* = 0.02) were associated with lower levels of depressive symptoms, while negative beliefs about preventative measures (β = 0.21, *p* < 0.001) predicted higher depressive symptoms. 

#### 3.3.3. Leisure Engagement and Current Mental Wellbeing

Similar to the results for perceived stress and depressive symptoms, the unadjusted model of mental wellbeing suggested that the frequency of leisure activities did not predict mental wellbeing, but the type of activity, change in engagement level, and evaluation of engagement were all significantly associated with mental wellbeing. After controlling for covariates, a much-less-than-ideal level of engagement remained a significant predictor of lower mental wellbeing (2.32 points lower than the about-ideal group, *p* = 0.001). 

The full model also suggested that life satisfaction (β = 0.50, *p* < 0.001) was the single strongest predictor of current mental wellbeing, followed by general physical health (β = 0.23, *p* < 0.001) and age (β = 0.21, *p* < 0.001). Four additional factors were significant, though with a smaller effect than a much-less-than-ideal engagement level: negative beliefs about preventative measures (β = −0.13, *p* < 0.001), being a female (β = −0.12, *p* = 0.001), and perceived risk of infection (β = −0.09, *p* = 0.016) were associated with lower levels of mental wellbeing, while people who were never married (β = 0.13, *p* = 0.006) reported better mental wellbeing than married people. Overall, leisure variables and the full model explained 8% and 51% of the variance in mental wellbeing, respectively.

## 4. Discussion

The present study investigated the broad pattern of leisure engagement among U.S. adults and its association with mental health and wellbeing at the height of the COVID-19 pandemic. The results revealed several important findings. 

### 4.1. What Is the Pattern of Leisure Engagement during COVID-19 and Its Association with Socio-Demographics?

Our results suggested that, while a very small proportion of people started a new favorite leisure activity during COVID-19, most people continued or fell back on activities they had engaged in before the pandemic, endorsing the value of established leisure activities through difficult times. Moreover, while the largest number of people identified a traditional leisure activity (e.g., reading, arts and crafts, or playing board games) as their favorite activity, a sizable share of (nearly one out of three) participants reported a digital/online activity (e.g., gaming or streaming) as their favorite. Although some of these activities could take place outside the home in a non-pandemic time (e.g., going to concerts in addition to listening to music at home, going out with family in addition to spending time together at home), people could easily maintain these activities in their reduced forms amid the restrictions throughout the pandemic. This is supported by the findings on engagement: people participated in most at-home traditional activities and all digital activities at high levels (close to 4–5 times a week) that generally exceeded corresponding pre-COVID levels. This finding is consistent with the ATUS result about the overall increased time for leisure among U.S. adults during the pandemic [34]. 

Importantly, our findings suggest that the increased leisure time was allocated unevenly across different activity domains. With the exception of walking and gardening, relatively low frequencies (less than 2–3 times a week) and reduced engagement (relative to the pre-COVID time) were reported for most physical and outdoor activities. This result was consistent with findings from previous studies that reported reduced physical activity and increased sedentary time in U.S. adults during the earlier stages of COVID-19 [39,41] and echoed findings from international studies [40,43,48,83]. The present study extended these findings to a later stage of the pandemic and to leisure pursuits more broadly by providing granular information on a diverse set of activities, allowing us to identify potential barriers to pandemic leisure. For example, although walking and gardening appeared less affected by the pandemic, these activities might not be an equalizer for those who lack access to a garden or a safe outdoor space for walking. Field/court sports and traveling saw the largest reduction in engagement during the pandemic, reflecting the effects of bans on large gatherings, widespread facility closures, and movement restrictions. While only a small proportion of respondents (<5%) identified these activities as their valued leisure, the changes these individuals experienced were likely steep and might induce a grave detrimental impact on their physical and mental health absent alternative leisure outlets.

Notably, nature-based activities were among the least participated activities (less than once a week). This finding contrasts the results of several studies conducted outside of the U.S. [52,84,85] and one U.S. study by Grima et al. [49], which all reported increased nature contact during COVID-19. This inconsistency can be partially reconciled by differences in timing and location of data collection. Most of these other studies were conducted shortly after the WHO declared COVID-19 a global pandemic when the lockdown effect was yet to become widespread [35]. In particular, Grima et al.’s study sample primarily consisted of regional existing park users, including onsite participants who already made it to a trailhead where they took the survey and were, therefore, likely biased towards regular park visitors or outdoor lovers who were less constrained by access to nature. Our results indicated that barriers to nature-based activities during the pandemic might be greater than previous studies suggested, and more research is needed to examine what factors might have posed constraints. Existing research in nature contact or outdoor recreation during the pandemic typically focused on access barriers or structural constraints [35]. More insights can be gained by looking into other types of constraints (e.g., intrapersonal and interpersonal constraints) [86] and corresponding adaptive behaviors that may inform intervention designs.

Our results on socio-demographic characteristics associated with different leisure domains revealed significant gender and age differences. These effects were controlled for in our regression analyses to ensure a more precise estimation of the association between leisure engagement and mental health. Notably, parent respondents reported significantly lower leisure participation than non-parenting participants, reflecting additional barriers to leisure among families with minors. According to the ATUS, although time spent in leisure activities increased for both individuals living in households with children and those in households without children, the former saw a smaller increase (25 vs. 36 min per day) and less overall leisure time (4.4 vs. 6.1 h per day) [34]. This gap is likely due to the increased time for childcare-related activities required of parenting individuals during COVID-19. As suggested by the ATUS, although adults living in households with children spent about the same time providing primary childcare in 2019 and 2020, they spent more time doing education-related activities for household children and providing secondary childcare during COVID-19, due to cancelations of children’s events and school closures or switching to virtual learning [34].

In summary, our study shed light on the broad pattern in American’s leisure engagement during the pandemic, expanding on leisure-related findings from large-scale, population-based surveys and previous studies with a narrower focus. Notable departures pertaining to nature-based leisure activities suggest opportunities for further research to uncover potential barriers to maximizing the benefit of nature contact in a time affected by drastic reduction in indoor leisure outlets. While endorsing the general trend of increased sedentary behavior and the need to tackle reduced physical activity in the population, the present study also calls for more attention to vulnerable minorities in the context of pandemic leisure, including (a) people who lack access to private gardens, safe and welcoming green spaces, or other resources that would facilitate engagement with nature and physical activity, (b) people with limited access to their current valued leisure activity or an alternative, and (c) parents who lack childcare support (also see [68]).

### 4.2. How Did Leisure Engagement Associate with Mental Health and Wellbeing?

After controlling for COVID-specific risk and protective factors, socio-demographic and COVID-related background variables, and general health and psychological wellbeing, frequency and leisure activity type did not predict perceived stress, but increased engagement (relative to pre-COVID level) was associated with higher levels of perceived stress. While causality cannot be inferred due to the cross-sectional nature of our data, this result supports a triggering effect, suggesting increasing engagement in valued leisure may be used as a compensatory strategy when people experience heightened stress. This is consistent with the detachment-recovery theory, which posits that individuals detach from stressors and seek recovery in leisure after periods of exerting efforts to address demands and stressors, including short-term exposure to precipitous stressful events and long-term exposure to continuous, evolving stressors without sufficient rest [87,88]. According to the Household Pulse Survey (HPS) by the U.S. Census Bureau, during 3–15 February 2021 (i.e., the time of this study), 39% of U.S. adults reported symptoms of anxiety and/or depressive disorder, and that percent had been largely stable since spring 2020. During the pandemic, as cumulative exposure to various COVID stressors causes increasing perceived stress, the need for recovery may motivate individuals to seek a much-needed respite in leisure to protect their wellbeing.

Findings from the depressive symptom model suggested that people experiencing reduced engagement in valued leisure reported significantly higher depressive symptoms than the maintenance group, while no differences were observed between the increased-engagement group and the maintenance group. This result complemented the detachment-recovery interpretation by revealing potential consequences of failing to maintain or increase leisure engagement under extended stress: people became vulnerable to increased risk of developing depression.

The results of the mental wellbeing model revealed slightly different findings on the predictors of positive mental health. After controlling for leisure activity type and frequency, people who felt their current engagement level fell far below their desired level reported significantly lower mental wellbeing compared to people whose level of engagement was close to ideal. Changes (increases or decreases) in engagement were not predictive of mental wellbeing. This result suggests that, different from the negative aspects of mental health (here perceived stress and depressive symptoms) that are closely associated with efforts aiming to address problems (e.g., increasing engagement to recover from stress) or indicators of being overcome by the adverse impact of stressors (e.g., reduced engagement due to the disruptions of COVID-19), mental wellbeing involves evaluating against qualitatively different references that shift the focus from problem-correction to optimal development and to hopes and ideals. This finding echoes the main proposition of the two-factor framework of resilience, which suggests that resilience is not just about compensating or rehabilitating, but also about attending to the positive for the purpose of enhancing well-being, and that the two factors likely further human development along independent trajectories [89]. Comparing the predictive power of leisure variables across the three regression models, our results demonstrated that (a) consistent with past research [10], individual leisure variables showed a moderate effect, and (b) collectively, leisure engagement was more strongly associated with mental wellbeing (R_j_^2^ = 8%) than with negative mental health (R_j_^2^ = 4% in perceived stress and depressive symptoms).

Integrating the above findings, the evidence from our study reinforces two important messages consistent with the findings of long-standing research on leisure and wellbeing and extend them to the current pandemic context: (a) leisure can be used as a resource toward alleviating psychological distress and furthering positive development, and (b) as a life domain that affords abundant opportunities for positive affective experiences that builds resources [90,91] and for fulfilling multiple higher-level psychological needs (e.g., autonomy, meaning, mastery, and affiliation) [18], leisure can be an important avenue for enhancing human well-being, even under a restrictive environment and sustained stress, such as that of COVID-19.

### 4.3. General Discussions and Implications

The results of this study also revealed findings on several important risk or protective factors of mental health in the context of COVID-19. Consistent with previous research, higher perceived risk of infection was a salient risk factor associated with higher stress and poorer mental health [1,2,4]. However, negative beliefs about public health measures (e.g., quarantine, masking, physical or social distancing, and closures) had a stronger impact on mental health and wellbeing (|β| = 0.13–0.25) than perceived risk of infection (|β| = 0.10). Beliefs about the negative and constraining effect of public health measures partly reflected the anxiety and stress that resulted from adhering to these restrictive prevention mandates that people might or might not identify with [1,92] and the broader negative pandemic impacts that were widely experienced across life domains [93]. Their stronger effect on mental health relative to the risk of infection itself contrasted findings from other countries [94] and mirrored the sentiment around the popular opposition of lives versus living that was exacerbated by the mixed messages from politicians and public health authorities since the U.S. entered the pandemic [95].

Our study provided evidence for the vulnerabilities of specific sectors of the U.S. population, including females, younger people, and those who perceived a poor financial condition, supporting findings from previous research [6,8]. Most notably, while a pre-existing condition was not significantly associated with any outcome measure in the full model, general physical health and long-term life satisfaction emerged as two strongest predictors of short-term mental health and wellbeing. This finding highlights the advantage and protective effect of maintaining optimal physical and mental health and, on the flip side, the vulnerabilities of those who suffer from long-term poor or sub-optimal health. The latter, while facing additional challenges associated with the pandemic (e.g., higher risk of infection due to compromised immune system and difficulty accessing health support and services [96]), lack the internal buffering resources that would help them weather through times of crisis and high stress.

The inclusion of the above salient risk and protective factors as covariates in our analysis is a strength of the present study, as previous studies typically accounted for a limited set of confounders. Moreover, the use of multiple measures of leisure engagement (i.e., variety, frequency, changes, and evaluations) and indicators of mental health (i.e., perceived stress, depressive symptoms, and mental wellbeing) allowed us to uncover some of the most revealing findings previously masked due to inadequate predictor and outcome measures and/or confounder control. Notably, although frequency has been commonly used to measure leisure engagement [22,23] and will likely remain useful in future surveys of leisure participation [10], our results suggested that in times of serious disruption and drastic deviation from the business-as-usual normal routine, measures that assess adaptive (e.g., increasing, reducing, or maintaining) engagement or a lack thereof may be a better predictor of health and wellbeing. Equally informative is the finding related to self-evaluation of engagement (relative to one’s desired level). Our results suggested that when positive mental health is the outcome of interest, attending to an individual’s own subjective evaluation of engagement, as opposed to imposing an external expert-determined standard for healthy engagement, has the potential to yield more valuable insight into participants’ wellbeing.

### 4.4. Limitations and Directions for Future Research

This research is not without limitations. Although our study sample was representative of the U.S. adult population in age, gender, race, and a number of key variables (e.g., vaccination rate, rate of working from home, and rate of depressive symptoms), it was not a random sample and did not represent the U.S. population in every aspect. Hence, we recommend caution in generalizing our results to the entire U.S. population. Nevertheless, the pattern of leisure engagement was consistent with statistics from national surveys of the same period (e.g., ATUS and HPS), speaking to the relatively high validity of our findings.

Second, there can be other ways to categorize leisure activities based on different criteria, including perceived functions [97], creativity [43], or level of socialization and structure. It would be interesting to see what insights can be uncovered by applying a different leisure categorization scheme. On a related note, because the aim of the present study was to characterize general patterns, we conducted group comparisons and regression analyses at the activity domain level but not at the lower activity category level. Future investigations may conduct more granular analyses of specific leisure categories or subcategories, which would require a larger sample to ensure adequate group sizes across categories.

Third, the sample size of the present study was modest relative to the number of covariates included in our regression models. While associations pertaining to key variables emerged from our analysis, a few relationships showed borderline significance, likely due to insufficient statistical power. Future research can benefit from the results of our covariate analyses by including a smaller set of covariates that is shown to strongly associate with the outcome variables without drastically increasing the sample size. Using a larger sample or not, careful considerations to minimize sample bias are warranted.

Finally, we noted two limitations related to the cross-sectional design of our study. (1) Seasonal effect was not accounted for in the present study, which might have biased or masked the findings related to physical/outdoor recreation to some extent [50]. (2) The cross-sectional nature of our data makes it challenging to establish causality. Including general health and long-term SWB as covariates in the regression helped to control for some of the confounding effect. However, to clearly determine the direction of the relationship between, for example, increased leisure engagement and higher perceived stress, would require an experimental or longitudinal design. Studies that employ a sequential explanatory design using mixed methods also have the potential to generate useful insights.

## 5. Conclusions

We identified broad patterns of leisure engagement among U.S. adults at the height of COVID-19, a period characterized by heightened stress, more time for leisure, and unprecedented disruptions and constraints. Our results suggested that, while there has been a general increase in leisure time during the pandemic, the additional time mostly went to home-based traditional leisure and digital or online activities. Physical and nature-based activities, on the other hand, saw reduced engagement, calling for further investigations on potential barriers that are likely greater than previously recognized. Our results also suggested that changes in engagement levels predicted negative mental health and subjective evaluation of engagement predicted mental wellbeing, above and beyond the effect of socio-demographics, COVID-specific risk and protective factors, and general health and long-term SWB. The results accentuated leisure’s dual role in facilitating stress alleviation and wellbeing enhancement in times of major disruptions and sustained stress.

At the time of the writing of this article, people around the world are still living with the evolving reality of the COVID-19 global pandemic. However, we want to conclude this article with a hopeful note: like every other epidemic in human history, this one will end. The long battle with the pandemic is a test of the functioning of our society and the strength of each individual. Despite the prolonged pandemic fatigue and the eagerness to turn the page, reflecting on what we have learned through this process will not only help us weather through the current pandemic, but also better manage the ones that will likely follow and future similar situations marked by sustained stress. A study on leisure engagement at the height of COVID-19 provides an opportunity to draw valuable lessons about our ability to adjust and adapt, to seek recovery, and to build resilience. Through illustrating some useful insights that can be derived by spotlighting leisure in taxing events, such as COVID-19, we hope to draw more attention to leisure’s potential as a framework for modeling both rehabilitation and optimal development toward resilience in health and wellbeing research.

## Figures and Tables

**Table 1:** Descriptive characteristics of socio-demographics and COVID-19 background variables (*n* = 503) ^1^.

Variable	*n*	%	M	*SD*
** Socio-demographics**				
Age ^2^	--	--	46.6	16.1
Gender				
Female	255	50.7		
Male	241	47.9		
Trans/non-binary	6	1.2		
Race				
White	370	73.7		
Asian	40	8.0		
Black/African American	70	13.9		
Other ^3^	22	4.4		
Ethnicity (Spanish, Hispanic, or Latino)				
No	463	92.8		
Yes	36	7.2		
Education attainment				
High school or less	51	10.2		
Some college/associate	165	32.9		
College	183	36.5		
Post-graduate	103	20.5		
Work status				
Work from home	214	42.8		
Work outside home: high-risk positions	36	7.2		
Work outside home: non-high-risk positions	56	11.2		
Unemployed/furloughed	176	35.2		
Household income				
<$29,999	121	24.1		
$30,000 to $69,999	197	39.2		
$70,000 to $99,999	92	18.3		
$100,000 or above	89	17.7		
Subjective financial conditions ^2^	--	--	3.4	1.1
Living area				
Rural/small town (<5000 population)	105	21.0		
Small city (5000–50,000)	137	27.4		
Medium/large city (>50,000 population)	258	51.6		
Marital status				
Married	223	44.6		
Never married	203	40.6		
Divorced/separated/widowed	74	14.8		
Having children				
No	308	61.6		
Yes	192	38.4		
**COVID-related background**				
Infection status				
No	435	87.7		
Yes, confirmed or suspected	61	12.3		
Vaccination status				
No	460	91.8		
Yes, partially vaccinated	33	6.6		
Yes, fully vaccinated	8	1.6		
Pre-existing conditions				
No	311	62.0		
Yes	191	38.0		

^1^*n* may vary across analyses due to missing responses. ^2^ Approximately linearly correlated with outcome variables. ^3^ Including Native Hawaiian/other pacific islander (*n* = 1), American Indian or Alaska Native (*n* = 6), and other (*n* = 15).

**Table 2:** Descriptive data on self-identified valued leisure activity (*n* = 502) ^1^.

Valued Leisure Activity	*n* (%)	Frequency (Past-Month)		Engagement Relative to Pre-COVID Level		Engagement Relative to Desired Level	
		M ^5^	(*SD*)	M ^5^	(*SD*)	M ^5^	(*SD*)
**Home-based offline activities**	**218 (43.4)**	**3.9 ^a^**	**1.30**	**3.3 ^a^**	**1.30**	**2.4 ^a^**	**0.79**
Reading and writing ^2^	94 (18.7)	4.1	1.18	3.3	1.31	2.4	0.72
Arts and crafts	36 (7.2)	3.3	1.25	3.2	1.30	2.2	0.80
Listening to music	17 (3.4)	4.4	1.00	3.7	0.92	2.7	0.59
Making music/singing/playing an instrument	14 (2.8)	3.4	1.55	2.5	1.29	1.7	0.91
Playing games offline	14 (2.8)	3.8	1.37	3.1	1.56	2.2	0.89
Spending time with family/friends	12 (2.4)	3.4	1.73	2.6	1.29	2.3	0.87
Cooking and baking	10 (2.0)	4.1	1.20	3.8	0.79	2.9	0.32
Relaxing ^3^	10 (2.0)	4.1	0.99	3.9	1.10	2.6	0.70
Other ^4^	10 (2.0)	--	--	--	--	--	--
**Screen-based digital/online activities**	**161 (32.1)**	**4.3 ^b^**	**1.07**	**3.73 ^b^**	**1.14**	**2.6 ^b^**	**0.65**
Playing video/online games	81 (16.1)	4.2	1.05	3.8	1.14	2.6	0.71
Watching TV/movies/videos	70 (13.9)	4.3	1.13	3.7	1.19	2.7	0.60
Computer/social media-based (non-gaming) activities	11 (2.2)	4.8	0.60	3.5	0.69	2.9	0.30
**Physical/outdoor activities**	**123 (24.5)**	**2.7 ^c^**	**1.55**	**2.6 ^c^**	**1.36**	**1.9 ^c^**	**0.87**
Exercising/working out	37 (7.4)	3.2	1.41	2.7	1.37	2.1	0.94
Nature-based activities	31 (6.2)	1.5	0.93	2.5	1.36	1.5	0.68
Walking	21 (4.2)	3.9	1.28	3.0	1.14	2.5	0.68
Gardening	13 (2.6)	3.8	1.41	3.0	1.29	2.1	0.86
Field/court sports	11 (2.2)	1.3	0.65	1.7	1.35	1.1	0.30
Traveling/driving	5 (1.0)	1.6	1.34	1.4	0.89	1.2	0.45
Skiing, rollerblading and board-sport	5 (1.0)	2.0	0.71	3.4	1.52	2.2	0.84

^1^ One participant did not provide information on leisure activity; ^2^ Including listening to audio books and podcast that did not involve extensive interactions with a screen; ^3^ examples include sleeping, taking baths, sitting outside, spending time with a pet; ^4^ Combining three categories of low occurrences (<5 or 1%): religious/spiritual practice, shopping, and home improvement/repairing; ^5^ Domain activity means with different letter superscripts in the same column differ significantly at *p* < 0.05 based on Kruskal–Wallis 1-way ANOVA pairwise comparisons.

**Table 3:** Valued leisure activities and the frequency of participation by socio-demographic characteristics.

	Type of Valued Leisure Activity				Level of Engagement (Frequency)	
Variable	Home-Based Offline Activities	Screen-Based Digital/Online Activities	Physical/ Outdoor Activities			
**Continuous variables**	M (*SD*) ^1^	M (*SD*) ^1^	M (*SD*) ^1^	*F*-value (*p*-value) Eta (η) ^2^	r (*p*-Value)	
Age	47.0 (16.3) ^ab^	43.8 (16.2) ^b^	49.7 (15.3) ^a^	**4.78 (0.009), 0.14**	0.33 (0.462)	
Subjective financial condition	3.36 (1.06)	3.28 (1.16)	3.38 (1.10)	2.29 (0.102)	0.01 (0.760)	
**Categorical variables**	*n* (row %)	*n* (row %)	*n* (row %)	*χ^2^* value (*p*-value) Cramer’s V^2^	M (*SD*)	*F*-value (*p*-value) Eta (η)^2^
Gender				**17.54 (<0.001),** **0.19**		3.63 (0.057)
Female	134 (52.5)	64 (25.1)	57 (22.4)		3.6 (1.5)	
Male	83 (34.6)	94 (39.2)	63 (26.3)		3.9 (1.4)	
Race/ethnicity				3.02 (0.554)		0.01 (0.986)
White	156 (42.3)	123 (33.3)	90 (24.4)		3.7 (1.5)	
Asian	16 (40.0)	10 (25.0)	14 (35.0)		3.8 (1.4)	
Black/African American	32 (45.7)	23 (32.9)	15 (21.4)		3.7 (1.4)	
Education attainment				**19.38 (0.004),** **0.14**		0.89 (0.447)
High school or less	17 (33.3)	23 (45.1)	11 (21.6)		4.0 (1.3)	
Some college/associate	65 (39.4)	67 (40.6)	33 (20.0)		3.7 (1.5)	
College	80 (43.7)	50 (27.3)	53 (29.0)		3.7 (1.5)	
Post-graduate	55 (53.9)	21 (20.6)	26 (25.5)		3.6 (1.4)	
Work status				10.56 (0.103)		2.13 (0.095)
Work from home	96 (44.9)	63 (29.4)	55 (25.7)		3.6 (1.5)	
Work outside home: high-risk positions	19 (52.8)	8 (22.2)	9 (25.0)		3.6 (1.3)	
Work outside home: non-high-risk positions	23 (41.1)	15 (26.8)	18 (32.1)		3.6 (1.5)	
Unemployed/furloughed	68 (38.6)	72 (40.9)	36 (20.5)		3.9 (1.4)	
Household income				**14.10 (0.029),** **0.12**		0.95 (0.417)
<$29,999	55 (45.5)	48 (39.7)	18 (14.9)		3.8 (1.4)	
$30,00 to $69,999	91 (46.2)	59 (29.9)	47 (23.9)		3.8 (1.4)	
$70,000 to $99,999	35 (38.0)	30 (32.6)	27 (29.3)		3.6 (1.6)	
$100,000 or above	34 (38.2)	24 (27.0)	31 (34.8)		3.6 (1.6)	
Living area				6.54 (0.162)		0.86 (0.425)
Rural/small town (<5000 population)	37 (35.2)	39 (37.1)	29 (27.6)		3.9 (1.3)	
Small city (5000–50,000)	70 (51.1)	40 (29.4)	27 (19.7)		3.7 (1.4)	
Medium/large city (>50,000 population)	110 (42.6)	82 (31.8)	66 (25.6)		3.7 (1.5)	
Marital status				5.99 (0.200)		1.43 (0.241)
Married	88 (39.5)	70 (31.4)	65 29.1)		3.7 (1.4)	
Never married	94 (46.5)	69 (34.2)	39 (19.3)		3.8 (1.4)	
Divorced/separated/widowed	34 (45.9)	22 (29.7)	18 (24.3)		3.5 (1.5)	
Parenting				1.24 (0.538)		**4.79 (0.029),** **0.10**
No	137 (41.1)	100 (31.8)	70 (27.1)		3.8 (1.4)	
Yes	79 (44.6)	61 (32.6)	52 (22.8)		3.5 (1.4)	

^1^ Means with different letter superscripts in the same row are significant at *p* < 0.05 based on Scheffe post-hoc tests for equal variances. ^2^ Effect size was calculated only if significant group differences existed (in bold).

**Table 4:** Fully adjusted linear regression models of perceived stress, depressive symptoms, and mental wellbeing (*n* = 467).

	Perceived Stress Model				Depressive Symptom Model				Mental Wellbeing Model			
	*B*	*SE*	β	∆R_j_^2^	*B*	*SE*	β	∆R_j_^2^	*B*	*SE*	β	∆R_j_^2^
Leisure engagement				0.04				0.04				0.08
Frequency	0.17	0.19	0.05		0.04	0.06	0.03		0.07	0.20	0.02	
Digital/online (Home-based non-digital = ref.)	−0.34	0.46	−0.03		−0.09	0.16	−0.02		−0.18	0.48	−0.01	
PA/outdoor (Home-based non-digital = ref.)	−0.35	0.51	−0.03		−0.06	0.17	−0.01		0.47	0.53	0.03	
Much less than pre-COVID (About same = ref.)	0.96	0.73	0.07		0.61*	0.25	0.12*		−0.89	0.77	−0.05	
Much more than pre-COVID (About same = ref.)	0.21 *	0.1	0.09 *		0.05	0.03	0.07		−0.06	0.10	−0.02	
Much less than ideal (About ideal = ref.)	1.19	0.68	0.10		0.19	0.23	0.05		−2.32 **	0.71	−0.16 **	
A little less than ideal (About ideal = ref.)	0.56	0.51	0.05		0.11	0.17	0.03		−1.03	0.54	−0.07	
COVID risk & protective factors				0.11				0.07				0.08
General risk of infection	0.39 *	0.16	0.10 *		0.01	0.05	0.01		−0.40 *	0.17	−0.09 *	
Safe health behavior	−0.29	0.25	−0.05		−0.06	0.09	−0.03		0.04	0.26	0.01	
Future outlook	0.31	0.19	0.07		0.11	0.06	0.07		0.08	0.19	0.01	
Positive beliefs about preventative measures	0.15	0.22	0.03		0.06	0.07	0.04		−0.05	0.23	−0.01	
Negative beliefs about preventative measures	1.00 ***	0.16	0.25 ***		0.28 ***	0.06	0.21 ***		−0.65 ***	0.17	−0.13 ***	
Socio-demographics & COVID background				0.18				0.13				0.08
Age	−0.13 ***	0.02	−0.44 ***		−0.03 ***	0.01	−0.33 ***		0.08 ***	0.02	0.21 ***	
Female (male = ref.)	0.46	0.4	0.05		0.16	0.14	0.05		−1.42 **	0.42	−0.12 **	
Subjective financial condition	−0.45 *	0.19	−0.10*		−0.12	0.06	−0.08		0.02	0.20	0.00	
Parenting (not parenting = ref.)	0.70	0.44	0.07		−0.15	0.15	−0.04		0.21	0.46	0.02	
Never married (married = ref.)	−0.67	0.56	−0.07		−0.44 *	0.19	−0.13 *		1.61 **	0.59	0.13 **	
Divorced/Separated/widowed (married = ref.)	−0.63	0.57	−0.05		−0.11	0.19	−0.02		0.70	0.60	0.04	
Infected (not infected = ref.)	1.09	0.59	0.07		0.30	0.20	0.06		−0.66	0.62	−0.04	
Pre-existing condition (no = ref.)	0.56	0.47	0.06		0.13	0.16	0.04		0.08	0.49	0.01	
General physical health and SWB				0.03				0.11				0.27
Physical health	−0.03 **	0.01	−0.15 **		−0.02 ***	0.00	−0.24 ***		0.06 ***	0.01	0.23 ***	
Life satisfaction	−0.06 *	0.03	−0.10 *		−0.05 ***	0.01	−0.23 ***		0.35 ***	0.03	0.50 ***	
Cumulative adjusted R^2^	0.36				0.35				0.51			
*F*-value	12.76				12.25				23.29			
F-test *p*-value	<0.001				<0.001				<0.001			

Notes: *B*: unstandardized coefficient; *SE*: standard error, β: standardized coefficient; ∆R_j_^2^: adjusted R-Square change; * *p*-value < 0.05; ** *p*-value < 0.01; *** *p*-value < 0.001.

## Data Availability

The data presented in this study are available on request from the corresponding author. The data are not publicly available due to ongoing analyses involving the data.

## Supplementary Materials

The following supporting information can be downloaded at: https://www.mdpi.com/article/10.3390/ijerph19031081/s1, Figure S1: Correlation matrix of the variables included in the adjusted multiple regression models (*n* = 467); Table S1: Descriptive data on socio-demographics, COVID-19 background variables, and their associations with mental health outcomes (*n* = 503); Table S2: Unadjusted and adjusted linear regression models of perceived stress (*n* = 467); Table S3: Unadjusted and adjusted linear regression models of depressive symptoms (*n* = 467); Table S4: Unadjusted and adjusted linear regression models of mental wellbeing (*n* = 467).
