# Supplementary material for: Leisure Engagement during COVID-19 and Its Association with Mental Health and Wellbeing in U.S. Adults
Source: Int J Environ Res Public Health. 2022 Jan 19;19(3):1081. doi: 10.3390/ijerph19031081 (PMC8834183; doi:10.3390/ijerph19031081)
Supplement: Supplementary file 1 [file ijerph-19-01081-s001.zip › ijerph-1537737-supplementary.pdf]

Supplemental material

Supplemental material Figure S1. Correlation matrix of the variables included in the adjusted multiple regression models (*n* = 467)

	Stress	Depression	EWB	favl_freq	Digital	PA_Outdoor	Change_Less	Change_More	Evl_Muchless	Evl_Littleless	COVID_RiskGeneral	COVID_Precaution	COV_Outlook	PosPM	NegPM	Age	Sex_dummy	SbjF	Parenting	NeverMarried	DSW	Infct	Pre_extn	physical_health	life_sat
Stress	--																								
Depression	0.666**	--																							
EWB	-0.525**	-0.636**	--																						
favl_freq	-0.047	-0.066	0.127**	--																					
Digital	0.033	0.033	-0.035	0.284**	--																				
PA_Outdoor	-0.097*	-0.078	0.098*	-0.411**	-0.391**	--																			
Change_Less	0.096*	0.165**	-0.201**	-0.561**	-0.229**	0.266**	--																		
Change_More	0.087	0.051	0.010	0.306**	0.199**	-0.174**	-0.222**	--																	
Evl_Muchless	0.092*	0.111*	-0.196**	-0.592**	-0.207**	0.284**	0.638**	-0.233**	--																
Evl_Littleless	0.054	0.027	-0.046	-0.110*	-0.098*	0.022	-0.137**	-0.150**	-0.287**	--															
COVID_RiskGeneral	0.168**	0.092*	-0.123**	0.008	-0.024	-0.055	0.009	0.138**	-0.009	-0.012	--														
COVID_Precaution	-0.100*	-0.061	0.015	0.026	0.000	-0.033	0.030	0.056	0.017	-0.103*	0.295**	--													
COV_Outlook	-0.021	-0.062	0.170**	-0.065	0.009	0.037	-0.024	-0.005	0.039	-0.001	0.080	0.128**	--												
PosPM	0.031	0.038	-0.020	0.043	-0.005	-0.098*	-0.023	0.044	-0.016	-0.040	0.354**	0.513**	0.255**	--											
NegPM	0.236**	0.216**	-0.170**	-0.092*	-0.076	0.098*	0.078	-0.033	0.070	0.115**	-0.095*	-0.207**	-0.055	-0.308**	--										
Age	-0.429*	-0.308**	0.216**	0.006	-0.121**	0.106*	0.051	-0.056	0.043	-0.066	-0.108*	0.199**	0.061	-0.045	0.009	--									
Sex_dummy	0.054	0.049	-0.125**	-0.086	-0.151**	-0.045	0.124**	0.018	0.043	-0.021	0.159**	0.182**	-0.146**	0.095*	-0.018	0.025	--								
SbjF	-0.248**	-0.271**	0.279**	-0.021	-0.063	0.091*	-0.027	-0.019	0.022	-0.014	-0.053	0.071	0.117**	0.018	-0.012	0.173**	-0.064	--							
Parenting	-0.048	-0.113*	0.094*	-0.098*	-0.008	0.048	0.083	-0.065	0.087	0.019	-0.042	-0.008	0.062	-0.108*	0.035	0.249**	0.053	0.094*	--						
NeverMarried	0.221**	0.176**	-0.178**	0.068	0.033	-0.099*	-0.028	0.066	-0.039	-0.037	0.101*	-0.056	-0.074	0.106*	-0.017	-0.542**	0.007	-0.221**	-0.510**	--					
DSW	-0.106*	-0.025	-0.016	-0.054	-0.023	-0.001	0.064	-0.067	0.027	0.013	-0.056	0.075	0.030	0.001	-0.018	0.244**	0.093*	-0.121**	0.123**	-0.345**	--				
Infct	0.159**	0.141**	-0.115*	0.001	0.007	-0.044	0.008	0.015	0.055	0.032	0.118**	-0.018	0.010	0.020	-0.015	-0.071	-0.035	-0.036	-0.002	-0.041	-0.035	--			
Pre_extn	0.098*	0.174**	-0.208**	0.006	0.024	-0.113*	0.133**	0.059	0.074	-0.044	0.132**	0.158**	-0.124**	0.096*	-0.017	0.214**	0.093*	-0.147**	0.004	-0.003	0.101*	0.095*	--		
physical_health	-0.258**	-0.382**	0.466**	-0.020	-0.091*	0.165**	-0.078	-0.029	-0.040	-0.005	-0.137**	-0.112*	0.166**	-0.138**	-0.057	-0.064	-0.012	0.236**	0.077	-0.064	-0.068	-0.167**	-0.503**	--	
life_sat	-0.257**	-0.396**	0.604**	-0.016	-0.092*	0.129**	-0.074	0.007	-0.044	0.002	0.029	0.046	0.201**	0.027	-0.069	0.130**	0.032	0.395**	0.186**	-0.306**	-0.085	-0.024	-0.190**	0.421**	--

** Correlation is significant at the 0.01 level (2-tailed)

* Correlation is significant at the 0.05 level (2-tailed)

Supplemental material Table S1. Descriptive data on socio-demographics, COVID-19 background variables, and their associations with mental health outcomes (N = 503)[1]

			Perceived stress			Depressive Symptoms			Mental Wellbeing		
Variable	*n* (%)	M(*SD*)	M(*SD*)[4]	*F*-value (*p*-value) Eta (ŋ)[5]	r (*p*-value)	M(*SD*)[4]	*F*-value (*p*-value) Eta (ŋ)[5]	r (*p*-value)	M(*SD*)[4]	*F*-value (*p*-value) Eta (ŋ)[5]	r (*p*-value
**Socio-demographics**											
Age[2]	--	46.6 (16.1)	--	--	**r = -0.43 (< 0.001)**	--	--	**r = -0.31 (< 0.001)**	--	--	**r = 0.22 (< 0.001)**
Gender				1.42			1.21			**7.86**	
Female	255 (50.7)		6.2 (5.1)	(0.233)		1.7 (1.7)	0(.272)		11.8 (6.1)[a]	**(0.005)**	
Male	241(47.9)		5.7 (4.9)			1.5 (1.6)			13.3 (5.8)[b]	**0.13**	
Trans/non-binary	6 (1.2)		--			--			--		
Race/ethnicity				0.55			1.46			1.24	
White	370 (73.7)		6.1 (4.9)	(0.577)		1.7 (1.7)	(0.233)		12.2 (5.8)	(0.291)	
Asian	40 (8.0)		5.8 (5.4)			1.5 (1.7)			13.7 (5.6)		
Black/African American	70 (13.9)		5.4 (5.4)			1.3 (1.6)			12.7 (6.8)		
Other[3]	22 (4.4)		--			--			--		
Education attainment				0.80			1.51[6]			2.35	
High school or less	51(10.2)		5.8 (4.7)	(0.493)		1.8 (1.6)	(0.214)		10.7 (5.4)	(0.072)	
Some college/associate	165 (32.9)		6.4 (5.1)			1.8 (1.9)			12.1 (5.9)		
College	183 (36.5)		5.6 (4.9)			1.4 (1.5)			12.7 (5.6)		
Post-graduate	103 (20.5)		6.2 (5.4)			1.5 (1.8)			13.2 (6.7)		
Work status				1.87			1.62			**2.68**	
Work from home	214 (42.8)		6.1 (5.3)	(0.134)		1.4 (1.6)	(0.183)		13.0 (6.3)	**(0.046)**	
Work outside home: high-risk positions	36 (7.2)		7.6 (4.8)			1.9 (1.5)			11.3 (5.2)	**0.13**	
Work outside home: non-high-risk positions	56 (11.2)		5.2 (4.3)			1.5 (1.7)			13.7 (6.0)		
Unemployed/furloughed	176 (35.2)		5.7 (4.8)			1.8 (1.8)			11.7 (5.7)		
Household income				1.04			2.59			**4.43**	
< $29,999	121 (24.1)		6.6 (4.8)	(0.374)		2.0 (1.8)	0(.052)		10.8 (5.4)[a]	**(0.004)**	
$30,000 to $69,999	197 (39.2)		5.6 (5.0)			1.5 (1.6)			12.8 (6.0)[b]	**0.16**	
$70,000 to $99,999	92 (18.3)		5.8 (5.0)			1.5 (1.7)			12.8 (5.8)[ab]		
$100,000 or above	89 (17.7)		6.4 (5.4)			1.5 (1.7)			12.4 (6.0)[b]		
Subjective financial conditions[2]	--	3.4 (1.1)	--	--	**r = -.24 (<0.001)**	--	**--**	**r = -.29 (<.001)**	--	**--**	**r =0 .29 (<0.001)**
Living area				2.14			0.14			0.10	
Rural/small town/town (<5,000 population)	105 (21.0)		5.8 (5.0)	(0.119)		1.6 (1.7)	(0.874)		12.5 (6.1)	(0.906)	
Small city (5,000 - 50, 000)	137 (27.4)		5.5 (4.9)			1.6 (1.7)			12.5 (6.2)		
Medium/large city (> 50,000 population)	258 (51.6)		6.5 (5.1)			1.7 (1.7)			12.3 (5.8)		
Marital status				**13.04 (<**			**8.31 (<**			**9.89**	
Married	223 (44.6)		5.3 (4.9)[a]	**0.001)**		1.3 (1.5)[a]	**0.001)**		13.6 (6.0)[a]	**(< 0.001)**	

Never married	203 (40.6)	7.4 (5.0)^{b}	**0.22**	2.0 (1.8)^{b}	**0.18**	11.1 (5.5)^{b}	**0.20**
Divorced/separated/widowed	74 (14.8)	4.8 (4.9)^{a}		1.5 (1.8)^{ab}		12.1 (6.3)^{a}	
Having children			1.14		**6.49**		**4.17**[6]
No	308 (61.6)	6.3 (5.0)	(0.285)	1.8 (1.7)	**(0.011)**	11.9 (5.6)	**(0.042)**
Yes	192 (38.4)	5.8 (5.0)		1.4 (1.7)	**0.11**	13.1 (6.4)	**0.09**
**COVID-related background**							
Infection status			**12.86**		**9.97**		**6.64**
No	435 (87.7)	5.7 (5.0)	**(<0 .001)**	1.5 (1.7)	**(0.002)**	12.7 (6.0)	**(0.01)**
Yes, confirmed or suspected	61(12.3)	8.1 (4.9)	**0.16**	2.3 (1.9)	**0.14**	10.6 (5.5)	**0.12**
Vaccination status			1.46		0.97		0.43
No	460 (91.8)	6.0 (5.1)	(0.235)	1.6 (1.7)	(0.378)	12.5 (6.0)	(.818)
Yes, partially vaccinated	33 (6.6)	6.2 (4.9)		2.0 (1.8)		11.7 (6.2)	
Yes, fully vaccinated	8 (1.6)	9.0 (3.3)		1.3 (1.4)		11.2 (7.02)	
Pre-existing conditions			**4.81**		**15.56**		**22.56**
No	311 (62.0)	5.6 (4.9)	**(0.029)**	1.4 (1.6)	**(<0.001)**	13.4 (5.8)	**(<0 .001)**
Yes	191 (38.0)	6.7 (5.2)	**0.10**	2.0 (1.8)	**0.17**	10.8 (6.0)	**0.21**

[1] *n* may vary across analyses due to missing responses. Numbers in bold indicate statistically significant results.

[2] Approximately lineally correlated with outcome variables

[3] Including Native Hawaiian/other pacific islander (n = 1), American Indian or Alaska Native (n = 6), and other (n = 15)

[4] Means with different letter superscripts in each row were significant at $p < 0.05$ based on Scheffe post-hoc tests for equal variances.

[5] Effect size was calculated only if significant group differences exist.

[6] Based on Welch statistic of robust testing for unequal variances

Supplemental material Table S2. Unadjusted and adjusted linear regression models of perceived stress (n = 467)

	Model 1			Model 2			Model 3			Model 4		
	B	*SE*	ß	*B*	*SE*	ß	*B*	*SE*	ß	*B*	*SE*	ß
Leisure engagement												
Frequency	0.08	0.22	0.02	0.07	0.21	0.02	0.19	0.19	0.05	0.17	0.19	0.05
Digital/online (Home-based non-digital = ref.)	0.20	0.54	0.02	0.46	0.51	0.04	-0.12	0.46	-0.01	-0.34	0.46	-0.03
PA/outdoor (Home-based non-digital = ref.)	-1.35	0.60	-0.12^{*}	-1.27	0.57	-0.11^{*}	-0.59	0.51	-0.05	-0.35	0.51	-0.03
Much less than pre-COVID (About same = ref.)	1.59	0.88	0.11	1.41	0.83	0.10	1.15	0.75	0.08	0.96	0.73	0.07
Much more than pre-COVID (About same = ref.)	0.33	0.11	0.14^{**}	0.26	0.11	0.11^{*}	0.20	0.10	0.08^{*}	0.21	0.10	0.09^{*}
Much less than ideal (About ideal = ref.)	1.71	0.82	0.14^{*}	1.48	0.77	0.12	1.34	0.69	0.11	1.19	0.68	0.10
A little less than ideal (About ideal = ref.)	1.60	0.61	0.14^{**}	1.00	0.58	0.08	0.72	0.52	0.06	0.56	0.51	0.05
COVID-specific risk and protective factors												
General risk of infection				0.73	0.18	0.19^{***}	0.39	0.16	0.10^{*}	0.39	0.16	0.10^{*}
Safe health behavior				-0.98	0.28	-0.18^{***}	-0.28	0.26	-0.05	-0.29	0.25	-0.05
Future outlook				-0.17	0.20	-0.04	0.13	0.18	0.03	0.31	0.19	0.07
Positive beliefs about preventative measures				0.54	0.24	0.12^{*}	0.26	0.22	0.06	0.15	0.22	0.03
Negative beliefs about preventative measures				1.07	0.18	0.26^{***}	1.10	0.16	0.27^{***}	1.00	0.16	0.25^{***}
Socio-demographics & COVID background												
age							-0.13	0.02	-0.42^{***}	-0.13	0.02	-0.44^{***}
Female (male = ref.)							0.24	0.40	0.02	0.46	0.40	0.05
Subjective financial condition							-0.67	0.18	-0.15^{***}	-0.45	0.19	-0.10^{*}
Parenting (not parenting = ref.)							0.57	0.45	0.06	0.70	0.44	0.07
Never married (married = ref.)							-0.34	0.55	-0.03	-0.67	0.56	-0.07
Divorced/Separated/widowed (married = ref.)							-0.41	0.58	-0.03	-0.63	0.57	-0.05
Infected (not infected = ref.)							1.42	0.59	0.09^{*}	1.09	0.59	0.07
Pre-existing condition (no = ref.)							1.28	0.43	0.13^{**}	0.56	0.47	0.06
General physical health & SWB												
Physical health										-0.03	0.01	-0.15^{**}
Life satisfaction										-0.06	0.03	-0.10^{*}
Adjusted R^2		0.04			0.15			0.33			0.36	
F-value		3.88			7.83			12.51			12.76	
F-test p-value		<0 .001			< 0.001			<0 .001			< 0.001	

Notes: *B*: unstandardized coefficient; *SE*: standard error, ß: standardized coefficient; * p-value < .05; ** p-value < .01; *** p-value < .001

Supplemental material Table S3. Unadjusted and adjusted linear regression models of depressive symptoms (n = 467)

	Model 1			Model 2			Model 3			Model 4		
	B	*SE*	ß	*B*	*SE*	ß	*B*	*SE*	ß	*B*	*SE*	ß
Leisure engagement												
Frequency	0.04	0.08	0.04	0.03	0.07	0.03	0.05	0.07	0.04	0.04	0.06	0.03
Digital/online (Home-based non-digital = ref.)	0.12	0.18	0.03	0.19	0.18	0.05	0.06	0.17	0.02	-0.09	0.16	-0.02
PA/outdoor (Home-based non-digital = ref.)	-0.45	0.20	-0.12*	-0.43	0.20	-0.11*	-0.21	0.18	-0.05	-0.06	0.17	-0.01
Much less than pre-COVID (About same = ref.)	0.92	0.30	0.19**	0.86	0.29	0.18**	0.73	0.27	0.15**	0.61	0.25	0.12*
Much more than pre-COVID (About same = ref.)	0.08	0.04	0.10*	0.06	0.04	0.08	0.05	0.03	0.06	0.05	0.03	0.07
Much less than ideal (About ideal = ref.)	0.40	0.28	0.10	0.33	0.27	0.08	0.29	0.25	0.07	0.19	0.23	0.05
A little less than ideal (About ideal = ref.)	0.44	0.21	0.11*	0.28	0.20	0.07	0.22	0.19	0.05	0.11	0.17	0.03
COVID-specific risk and protective factors												
General risk of infection				0.11	0.06	0.08	0.01	0.06	0.01	0.01	0.05	0.01
Safe health behavior				-0.22	0.10	-0.12*	-0.05	0.09	-0.03	-0.06	0.09	-0.03
Future outlook				-0.11	0.07	-0.07	-0.01	0.07	-0.01	0.11	0.06	0.07
Positive beliefs about preventative measures				0.21	0.08	0.14*	0.12	0.08	0.08	0.06	0.07	0.04
Negative beliefs about preventative measures				0.33	0.06	0.24***	0.34	0.06	0.25***	0.28	0.06	0.21***
Socio-demographics & COVID background												
age							-0.03	0.01	-0.30***	-0.03	0.01	-0.33***
Female (male = ref.)							0.02	0.14	0.01	0.16	0.14	0.05
Subjective financial condition							-0.27	0.07	-0.18***	-0.12	0.06	-0.08
Parenting (not parenting = ref.)							-0.24	0.16	-0.07	-0.15	0.15	-0.04
Never married (married = ref.)							-0.20	0.20	-0.06	-0.44	0.19	-0.13*
Divorced/Separated/widowed (married = ref.)							0.06	0.21	0.01	-0.11	0.19	-0.02
Infected (not infected = ref.)							0.50	0.21	0.10*	0.30	0.20	0.06
Pre-existing condition (no = ref.)							0.55	0.15	0.16***	0.13	0.16	0.04
General physical health & SWB												
Physical health										-0.02	0.00	-0.24***
Life satisfaction										-0.05	0.01	-0.23***
Adjusted R^2	0.04			0.11			0.24			0.35		
F-value	4.11			5.85			8.54			12.25		
F-test *p*-value	< 0.001			<0 .001			< 0.001			< 0.001		

Notes: *B*: unstandardized coefficient; *SE*: standard error, ß: standardized coefficient; * *p*-value < .05; ** *p*-value < .01; *** *p*-value < .001

Supplemental material Table S4. Unadjusted and adjusted linear regression models of mental wellbeing (*n* = 467)

	Model 1			Model 2			Model 3			Model 4		
	B	*SE*	ß	*B*	*SE*	ß	*B*	*SE*	ß	*B*	*SE*	ß
Leisure engagement												
Frequency	-0.06	0.26	-0.01	0.02	0.25	0.00	-0.01	0.24	0.00	0.07	0.20	0.02
Digital/online (Home-based non-digital = ref.)	-0.77	0.63	-0.06	-1.10	0.61	-0.09	-0.91	0.59	-0.07	-0.18	0.48	-0.01
PA/outdoor (Home-based non-digital = ref.)	2.23	0.70	0.16**	2.05	0.68	0.15**	1.28	0.66	0.09	0.47	0.53	0.03
Much less than pre-COVID (About same = ref.)	-2.61	1.03	-0.15*	-2.17	0.99	-0.12*	-1.61	0.95	-0.09	-0.89	0.77	-0.05
Much more than pre-COVID (About same = ref.)	-0.12	0.13	-0.04	-0.06	0.13	-0.02	-0.02	0.12	-0.01	-0.06	0.10	-0.02
Much less than ideal (About ideal = ref.)	-3.18	0.96	-0.22**	-3.09	0.92	-0.21**	-3.02	0.88	-0.21**	-2.32	0.71	-0.16**
A little less than ideal (About ideal = ref.)	-2.15	0.72	-0.15**	-1.72	0.70	-0.12*	-1.61	0.66	-0.11*	-1.03	0.54	-0.07
COVID-specific risk and protective factors												
General risk of infection				-0.59	0.21	-0.13**	-0.31	0.21	-0.07	-0.40	0.17	-0.09*
Safe health behavior				0.36	0.33	0.06	0.02	0.33	0.00	0.04	0.26	0.01
Future outlook				1.05	0.24	0.20***	0.69	0.24	0.13**	0.08	0.19	0.01
Positive beliefs about preventative measures				-0.54	0.29	-0.10	-0.27	0.28	-0.05	-0.05	0.23	-0.01
Negative beliefs about preventative measures				-0.93	0.22	-0.19***	-0.94	0.21	-0.19***	-0.65	0.17	-0.13***
Socio-demographics & COVID background												
age							0.06	0.02	0.17**	0.08	0.02	0.21***
Female (male = ref.)							-0.61	0.52	-0.05	-1.42	0.42	-0.12**
Subjective financial condition							0.93	0.23	0.17***	0.02	0.20	0.00
Parenting (not parenting = ref.)							0.63	0.57	0.05	0.21	0.46	0.02
Never married (married = ref.)							-0.19	0.71	-0.02	1.61	0.59	0.13**
Divorced/Separated/widowed (married = ref.)							-0.46	0.74	-0.03	0.70	0.60	0.04
Infected (not infected = ref.)							-1.45	0.76	-0.08	-0.66	0.62	-0.04
Pre-existing condition (no = ref.)							-1.67	0.55	-0.14**	0.08	0.49	0.01
General physical health & SWB												
Physical health										0.06	0.01	0.23***
Life satisfaction										0.35	0.03	0.50***
Adjusted R^2	0.08			0.16			0.24			0.51		
F-value	7.15			8.45			8.58			23.29		
F-test *p*-value	<0.001			< 0.001			< 0.001			< 0.001		

Notes: *B*: unstandardized coefficient; *SE*: standard error, ß: standardized coefficient; * *p*-value < .05; ** *p*-value < .01; *** *p*-value < .001
